# RACE AND GENDER DIFFERENCES IN UNMET NEEDS OF NONSPOUSAL CARE PARTNERS OF PEOPLE LIVING WITH DEMENTIA

**DOI:** 10.1093/geroni/igae098.0590

**Published:** 2024-12-31

**Authors:** Gretchen Tucker, Ann Gruber-Baldini, Quincy Samus, Laura Girling, Denise Orwig

**Affiliations:** University of Maryland Baltimore, Baltimore, Maryland, United States; University of Maryland School of Medicine, Baltimore, Maryland, United States; Johns Hopkins University, Baltimore, Maryland, United States; Towson University, Towson, Maryland, United States; University of Maryland School of Medicine, Baltimore, Maryland, United States

## Abstract

Research on unmet care needs of non-spousal informal care partners (ICPs) for persons living with Alzheimer’s disease and related dementias (P-ADRD) is limited. A secondary data analysis using linear and logistic regressions was employed to explore non-spousal ICPs’ unmet needs by gender and race using the Johns Hopkins Dementia Care Needs Assessment (JHDCNA 2.0©). The JHDCNA 2.0© includes six domains and 18 items assessing ICPs’ needs. Data included 413 ICPs of P-ADRD from two community-based studies: Maximizing Independence at Home Randomized Control Trial and Maximizing Independence at Home-Health Care Innovation Award, which enrolled dyads of P-ADRD and their ICPs. Of the 413 ICPs, 143 (34.6%) were White, 270 (65.4%) Black, with 343 (83.1%) female, and 70 (16.9%) male. Gender was not significantly associated with overall percentage of unmet dementia-related care needs. However, there was a race by sex interaction (p <.001); Black male non-spousal ICPs had the highest mean total percent unmet needs (33.48±13.87) while White female non-spousal ICPs had the lowest mean total percent of unmet needs (28.03±13.75). Being a Black non-spousal ICPs was associated with having higher odds of having unmet needs in specific categories such as behaviors 73% vs 61% (p =.015), substitute decision-making 29% vs 8% (p <.001), and decision-making documents 84% vs 71% (p =.002) compared to White non-spousal ICPs.This study highlights the importance of understanding the unique needs of non-spousal ICPs, particularly differences by race, to ensure access to resources and assistance for persons providing care to individuals living with ADRD.

